# Clinical efficacy and pregnancy outcomes of doxycycline plus medroxyprogesterone acetate therapy for chronic endometritis in women with recurrent reproductive failure: a prospective cohort study

**DOI:** 10.3389/fmed.2026.1913511

**Published:** 2026-09-24

**Authors:** Fei Yang, Dongrui Peng, Chen Wang, Xin Yang, Lijie Wang, Yaolong Chen, Fang Wang

**Affiliations:** 1 Department of Reproductive Medicine, The Second Hospital of Lanzhou University, Lanzhou, People's Republic of China; 2 School of Public Health, Lanzhou University, Lanzhou, People's Republic of China

**Keywords:** chronic endometritis, doxycycline, medroxyprogesterone acetate, prospective cohort study, recurrent reproductive failure

## Abstract

**Introduction:**

Chronic endometritis (CE) is associated with recurrent reproductive failure (RRF), but the optimal treatment strategy remains uncertain. This study evaluated the efficacy and safety of doxycycline plus medroxyprogesterone acetate (MPA) in women with RRF and CE.

**Material and methods:**

This prospective cohort study included 330 women with RRF and CE diagnosed by CD138 immunohistochemistry. According to the treatment received, patients were classified into the doxycycline group or the doxycycline plus MPA group, with 165 women in each group. The primary outcome was the histological cure rate of CE after one treatment course. Secondary outcomes included pregnancy rate, live birth rate, pregnancy loss rate, ongoing pregnancy rate, and adverse events. Multivariable Poisson regression with robust variance was used to adjust for potential confounders.

**Results:**

In the primary efficacy population, CE cure occurred in 117 of 158 patients (74.1%) receiving doxycycline and 130 of 160 (81.2%) receiving doxycycline plus MPA [adjusted risk difference [aRD], 6.1; 95% CI, −3.2–15.4; adjusted risk ratio [aRR], 1.08; 95% CI, 0.96–1.22; *P* = 0.202]. Pregnancy occurred in 97 of 158 patients (61.4%) and 126 of 160 (78.8%) (aRD, 16.5 percentage points; 95% CI, 6.5–26.5; aRR, 1.27; 95% CI, 1.09–1.47; *P* = 0.002). Adverse events occurred in 12 of 165 patients (7.3%) in each group and were all mild.

**Conclusion:**

Doxycycline plus MPA did not significantly improve the CE cure rate in women with RRF and CE, but was associated with a higher pregnancy rate without an increase in adverse events.

**Trial registration:**

This study was registered in the Chinese Clinical Trial Registry with the registration number of ChiCTR2400080514 (31/01/2024)

## Introduction

Recurrent reproductive failure (RRF), including recurrent pregnancy loss (RPL) and recurrent implantation failure (RIF), remains a major challenge in reproductive medicine (1). The condition is multifactorial and may substantially affect patients' physical and psychological well-being (2). The endometrium plays a central role in implantation and early pregnancy through its support of blastocyst adhesion, trophoblast invasion, and fetal development (3). Among endometrial disorders, chronic endometritis (CE) has attracted increasing attention because of its association with adverse reproductive outcomes in women with RRF (4). Reported prevalence estimates of CE range from 14% to 67.5% in women with RIF and from 9.3% to 67.6% in women with RPL (5–7).

CE is a persistent inflammatory condition of the endometrium, characterized histologically by stromal edema, increased stromal cell density, and plasma cell infiltration (8, 9). It is often asymptomatic or associated with only mild symptoms, such as abnormal uterine bleeding, pelvic pain, or abnormal vaginal discharge (10). CE is commonly associated with pathogenic infection, and frequent causative organisms include Streptococcus, Enterococcus faecalis, Escherichia coli, Staphylococcus species, Ureaplasma urealyticum, and Chlamydia (11). Therefore, empirical treatment with broad-spectrum antibiotics remains the main therapeutic approach for CE.

Doxycycline, a tetracycline-class broad-spectrum antibiotic, is widely regarded as safe and effective in the treatment of pelvic inflammatory disease and is therefore one of the most commonly used antibiotics for CE. Previous studies have reported a single-course cure rate ranging from 66.6% to 92.3% (12–15). Although antibiotic therapy can improve the cure rate of CE, its impact on subsequent reproductive outcomes remains inconsistent. Gay et al. found that, compared with untreated patients, women with CE who received 14 days of antibiotic therapy had a significantly higher live birth rate (LBR) (13). A systematic review and meta-analysis showed that, in women with RIF, antibiotic treatment did not confer significant benefits in terms of ongoing pregnancy rate (OPR), LBR, clinical pregnancy rate (CPR), or implantation rate (IR); however, patients with cured CE had higher OPR, LBR, CPR, and IR than those with persistent CE (16).

Recently, a randomized controlled trial suggested that dydrogesterone combined with doxycycline was superior to doxycycline alone in achieving CE cure, possibly because dydrogesterone promotes secretory transformation of the endometrium and exerts immunomodulatory effects (17). Combined therapy may therefore improve endometrial status and increase pregnancy rates while controlling infection, particularly in women with CE-related infertility (18).

Compared with dydrogesterone, medroxyprogesterone acetate (MPA) has stronger progestational activity and may improve the embryo implantation environment by enhancing endometrial secretory function. In addition, MPA has glucocorticoid-like immunomodulatory properties and has been used in some inflammatory and autoimmune disorders (19, 20). Previous studies have shown that MPA may promote monocyte differentiation toward the M2 phenotype, facilitate decidualization of endometrial stromal cells, and enhance trophoblast invasion (21). However, clinical evidence on whether doxycycline combined with MPA improves CE cure and reproductive outcomes in women with RRF is lacking. Therefore, we conducted a prospective cohort single-center study to evaluate the efficacy and safety of doxycycline plus MPA in women with RRF-associated CE.

## Materials and methods

### Participants and ethics

This prospective single-center cohort study included women who attended the Reproductive Medicine Center of Lanzhou University Second Hospital, met the diagnostic criteria for RRF, and were diagnosed with CE by endometrial CD138 immunohistochemical staining. Baseline data were collected before treatment. Patients were classified into the doxycycline group or the doxycycline plus MPA group according to the treatment actually received in clinical practice.

The inclusion criteria were as follows: 1) age 18–45 years, planning pregnancy within the following 12 months, and diagnosed with CE by endometrial CD138 immunohistochemical staining; 2) meeting the diagnostic criteria for RRF [RPL is typically defined as the loss of two or more pregnancies before 24 weeks of gestation (22); RIF as the failure to achieve a clinical pregnancy after two to three transfers with good-quality embryos (23); 3] willingness to undergo hysteroscopy and endometrial biopsy; and 4) no contraindications to doxycycline or MPA.

The exclusion criteria were as follows: 1) acute genital tract infection, gynecological malignancy, endometrial hyperplasia, or uterine fibroids; 2) pregnancy or lactation; 3) contraindications to hysteroscopy, hysteroscopic surgery, or anesthesia; 4) use of anticoagulant, immunomodulatory, or hormonal medications within the preceding 3 months; 5) history of allergy to doxycycline or MPA; and 6) documented azoospermia in the male partner (defined as the absence of spermatozoa in the ejaculate, based on a semen-analysis report issued by our hospital or another medical institution (24).

The study protocol was approved by the Ethics Committee of Lanzhou University Second Hospital (2024A-030) and registered in the Chinese Clinical Trial Registry (ChiCTR2400080514). All patients provided written informed consent before enrollment.

### Sample size calculation

Before patient recruitment, the sample size was calculated using PASS version 11.0 (NCSS, Kaysville, UT, USA) for the comparison of two independent proportions. Based on the available comparative evidence, the anticipated cure rates were 86.0% in the doxycycline plus dydrogesterone group and 72.6% in the doxycycline-only group (17), corresponding to an absolute difference of 13.4 percentage points and a relative risk of 1.18. This assumed difference was used for sample size planning and was not prespecified as a minimum clinically important difference. Using a two-sided unpooled *Z* test without continuity correction, with an *α* level of 0.05, 80% power, and an anticipated 1:1 group-size ratio, 140 evaluable patients were required in each group. The unpooled procedure was selected *a priori* because the calculation was based on distinct anticipated cure rates for the two groups and therefore used the corresponding group-specific binomial variances under the alternative hypothesis. After allowing for a 15% rate of loss to follow-up or missing primary outcome data, the planned recruitment target was 165 patients per group, for a total of 330 patients.

### Treatment exposure and grouping

After CE was diagnosed, all patients received treatment recommendations from clinicians according to routine clinical practice. The doses of doxycycline and MPA and the 14-day treatment duration were selected with reference to the relevant Chinese prescribing information and the short-course regimens routinely used at our center. Both treatment regimens were initiated on menstrual cycle day 15 to standardize treatment timing across the two groups and to ensure the simultaneous initiation of doxycycline and MPA in the combination group. This timing was also selected because withdrawal bleeding may occur after MPA discontinuation, allowing such bleeding to occur around the expected onset of the subsequent menstrual period. Treatment decisions were made by physicians based on patients' clinical characteristics, previous medication history, menstrual cycle status, contraindications, and patient preference. The investigators did not interfere with treatment selection, but prospectively recorded treatment exposure, post-treatment CE status, adverse events, and subsequent reproductive outcomes. According to the treatment actually received, patients were divided into two groups:

(1) Doxycycline group: oral doxycycline 100 mg twice daily for 14 consecutive days.
(2) Doxycycline plus MPA group: oral doxycycline 100 mg twice daily combined with oral MPA 8 mg three times daily for 14 consecutive days.

### Hysteroscopy and endometrial biopsy

All patients underwent hysteroscopy 3–7 days after menstruation. Hysteroscopy was performed with a 3-mm 30° rigid hysteroscope (Olympus) with an outer diameter of 4.5 mm by one of two experienced specialists who were blinded to the results of histologic assessment regarding CE. Normal saline solution was used to distend the uterine cavity at 100 mmHg pressure. After hysteroscopy, an endometrial biopsy specimen was obtained with the use of a metal curette from the upper uterine cavity.

### Histology and immunohistochemistry

Endometrial samples were fixed in formalin, embedded in paraffin, and subjected to routine hematoxylin and eosin staining and CD138 immunohistochemical staining. Plasma cells were identified using hematoxylin and eosin staining combined with CD138 immunohistochemistry. CE was diagnosed when ≥1 plasma cell was detected in every 10 high-power fields (HPFs) of the endometrial stroma (25–27). Mild CE was defined as 1–4 plasma cells per 10 HPFs, whereas severe CE was defined as ≥5 plasma cells per 10 HPFs (28, 29). Repeat hysteroscopy and endometrial biopsy were performed on menstrual cycle days 3–7 in the next cycle to assess treatment response. All specimens and repeat endometrial specimens were reviewed by a single experienced pathologist who was blinded to treatment allocation and follow-up reproductive outcomes. The same predefined histopathological criteria were applied to both baseline and repeat specimens. Histopathological reports were typically available within 3–7 days after sampling.

### Procedures and outcome definitions

At screening and enrollment, trained members of the study team (FY and DP) prospectively collected baseline demographic and reproductive information using a standardized case report form. Data were obtained through face-to-face interviews and review of the electronic medical records, including age, body mass index, previous pregnancy history, previous delivery history, number of previous pregnancy losses, history of pelvic surgery, RRF subtype, and CE severity.

After CE was confirmed and the histopathological result was communicated, the treatment regimen was selected during routine clinical care. Both treatment regimens were initiated on menstrual cycle day 15 and prescribed for 14 consecutive days. Treatment adherence was assessed by patient self-report during telephone or outpatient follow-up. patients were asked whether they had completed the prescribed 14-day treatment course and whether any doses had been missed. Self-discontinuation before completion of the prescribed course was recorded as non-adherence. After treatment, patients were scheduled to undergo repeat hysteroscopy and endometrial biopsy during the follicular phase of the subsequent menstrual cycle to assess chronic endometritis cure. Adverse events were recorded during treatment and follow-up, including gastrointestinal discomfort, dizziness, allergic reactions, hepatic or renal dysfunction, and coagulation abnormalities.

Reproductive outcomes were followed up every 3 months for up to 12 months through outpatient visits or telephone interviews conducted by trained study personnel (FY and DP). Data were collected on pregnancy occurrence, mode of conception, pregnancy continuation, pregnancy loss, ongoing pregnancy, and live birth. For patients with more than one pregnancy during follow-up, only the outcome of the first pregnancy was included in the analysis. Pregnancy was defined as a positive serum or urinary *β*-human chorionic gonadotropin test (30). Ongoing pregnancy was defined as a pregnancy continuing for at least 12 weeks (28). Live birth was defined as delivery at or after 24 weeks of gestation with signs of life (31). Pregnancy loss was defined as spontaneous pregnancy loss before 24 weeks of gestation (28).

Adverse events were defined as any unfavorable medical events occurring during treatment or follow-up, regardless of whether a definite causal relationship with treatment was established. All adverse events were collected through patient self-report, outpatient medical records, and follow-up interviews. The time of onset, severity, duration, management, and outcome were recorded. Serious adverse events, if any, were managed promptly and reported according to the requirements of the ethics committee.

### Statistical methods

The primary efficacy analysis included patients who completed the prescribed treatment and underwent post-treatment endometrial pathological reassessment. The safety analysis included all patients who received at least one dose of treatment and had safety records available. The primary reproductive-outcome analysis included all patients entering reproductive follow-up. patients with incomplete reproductive follow-up were conservatively classified as not having experienced the corresponding outcome. A complete-case sensitivity analysis was performed among patients with complete reproductive follow-up. To assess the robustness of the findings to missing post-treatment cure assessments and incomplete treatment, a conservative full-cohort sensitivity analysis was performed. All enrolled patients were analyzed according to the treatment regimen initially initiated.

Patients who did not complete the scheduled post-treatment hysteroscopic and endometrial histopathological assessment or who discontinued the prescribed 14-day treatment course before completion were classified as not cured. patients meeting both criteria were counted only once. No other missing outcome values were imputed.

Given the observational design, covariates were selected before outcome modeling on the basis of clinical relevance and previous evidence, rather than between-group statistical significance in Table 1. The adjustment set included age, body mass index, baseline CE severity, number of previous live births, number of previous pregnancy losses, history of pelvic surgery, and reproductive failure subtype (recurrent pregnancy loss or recurrent implantation failure). Unadjusted and adjusted risk differences were estimated using linear probability models with HC1 robust sandwich variance estimators, whereas unadjusted and adjusted risk ratios were estimated using modified Poisson regression models with a log link and HC1 robust sandwich variance estimators. Robust variance estimation was used because the conventional Poisson mean–variance assumption is not appropriate for binary outcomes. The same adjustment set was used for all main adjusted efficacy analyses. In analyses stratified by baseline CE severity, CE severity was omitted because it had no within-stratum variation. Model diagnostics included assessment of convergence, design-matrix rank deficiency, and whether fitted means were finite and positive effect estimates were reported with 95% confidence intervals. All tests were two-sided, and *P* < 0.05 was considered statistically significant. Statistical analyses were performed using R software, version 4.5.2 (R Foundation for Statistical Computing; regression packages: stats, sandwich, and lmtest).

**Table 1:** Baseline characteristics.

Characteristic	Doxycycline Group (*n* = 165)	Doxycycline plus MPA Group (*n* = 165)	P
Age, years (mean ± SD)	31.33 ± 4.70	30.94 ± 4.49	0.444
BMI, kg/m^2^ (mean ± SD)	23.40 ± 3.38	22.80 ± 3.14	0.098
CE severity, n (%)			0.822
Mild CE	98 (59.4%)	101 (61.2%)	
Severe CE	67 (40.6%)	64 (38.8%)	
Previous history of pelvic surgery, n (%)			0.561
No	106 (64.2%)	112 (67.9%)	
Yes	59 (35.8%)	53 (32.1%)	
RRF subtype, n (%)			0.685
RPL	161 (97.6%)	163 (98.8%)	
RIF	4 (2.4%)	2 (1.2%)	
Previous live births, n (%)			0.422
0	140 (84.8%)	139 (84.2%)	
1	22 (13.3%)	19 (11.5%)	
2	3 (1.8%)	7 (4.2%)	
Number of previous pregnancy losses (%)			0.706
2	154 (93.3%)	151 (91.5%)	
3	7 (4.2%)	11 (6.7%)	
≥ 4	4 (2.4%)	3 (1.8%)	

*Data are presented as mean ± SD or n (%). BMI, body mass index; CE, chronic endometritis; MPA, medroxyprogesterone acetate; RRF, recurrent reproductive failure; RPL, recurrent pregnancy loss; RIF, recurrent implantation failure.

## Results

### Patient characteristics

Between April 2024 and July 2025, 429 patients with confirmed CE were assessed for eligibility. Of these, 99 were excluded: 98 did not meet the prespecified criteria for RRF, and one declined to participate. 330 patients were enrolled in the prospective cohort. According to the treatment actually received, patients were classified into the doxycycline group and the doxycycline plus MPA group, with 165 patients in each group. Of the 330 patients, 12 patients were not included in the primary efficacy analysis, including 7 who did not complete the second hysteroscopy and endometrial biopsy after treatment and 5 who did not complete the prescribed treatment course (Figure 1). Therefore, 318 patients were included in the primary efficacy analysis, including 158 in the doxycycline group and 160 in the doxycycline plus MPA group. Analysis-population membership and missing outcome data are summarized by treatment group in Supplementary Table S1. Among these 318 patients, 306 completed reproductive follow-up, whereas 12 had incomplete follow-up. No additional patients were excluded from the adjusted analyses because of missing prespecified covariates. Baseline demographic and clinical characteristics are shown in Table 1. There were no statistically significant differences between the two groups in age, body mass index, number of previous live births, number of previous pregnancy losses, or history of pelvic surgery (all *P* > 0.05).

**Figure 1:**
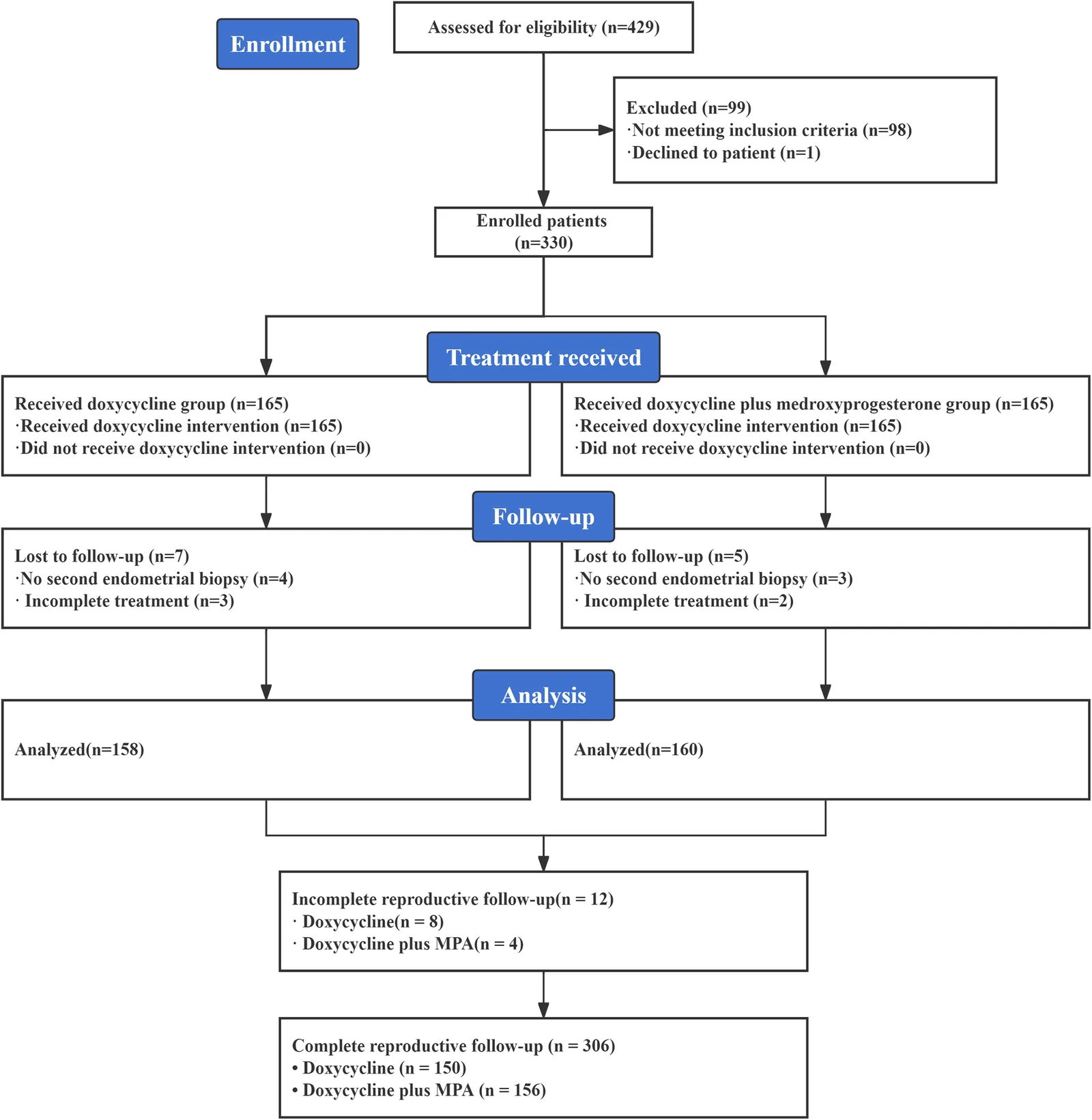
Study flow chart.

### Treatment outcomes for CE

CE cure was defined as the absence of CD138-positive plasma cells on post-treatment endometrial histological examination. The primary efficacy analysis included 318 patients who completed the prescribed treatment and underwent post-treatment pathological reassessment. CE cure occurred in 130 of 160 patients (81.2%) receiving doxycycline plus MPA and 117 of 158 patients (74.1%) receiving doxycycline alone. The adjusted estimates did not provide evidence of a between-group difference(    aRD, 6.1 percentage points, 95% CI, −3.2–15.4;   aRR, 1.08; 95% CI, 0.96–1.22; *P* = 0.202) (Table 2).

**Table 2:** Clinical outcomes according to treatment regimen.

Outcome	Doxycycline	Doxycycline + MPA	RD, percentage points (95% CI); P	RR (95% CI); P	aRD, percentage points (95% CI); P	aRR (95% CI); P
CE outcomes						
CE cure, primary efficacy analysis	117/158 (74.1%)	130/160 (81.2%)	7.2 (−2.0 to 16.4); 0.124	1.10 (0.97 to 1.24); 0.126	6.1 (−3.2 to 15.4); 0.197	1.08 (0.96 to 1.22); 0.202
CE cure, conservative full-cohort sensitivity analysis	117/165 (70.9%)	130/165 (78.8%)	7.9 (−1.5 to 17.2); 0.100	1.11 (0.98 to 1.26); 0.102	6.0 (−3.4 to 15.4); 0.211	1.08 (0.95 to 1.23); 0.214
Mild CE	75/96 (78.1%)	81/98 (82.7%)	4.5 (−6.7 to 15.7); 0.430	1.06 (0.92 to 1.22); 0.431	4.5 (−6.7 to 15.8); 0.431	1.06 (0.92 to 1.22); 0.440
Severe CE	42/62 (67.7%)	49/62 (79.0%)	11.3 (−4.3 to 26.8); 0.157	1.17 (0.94 to 1.45); 0.162	11.2 (−6.3 to 28.7); 0.212	1.17 (0.92 to 1.48); 0.213
Reproductive outcomes, primary analysis						
Pregnancy	97/158 (61.4%)	126/160 (78.8%)	17.4 (7.4 to 27.3); < .001	1.28 (1.11 to 1.49); < .001	16.5 (6.5 to 26.5); .001	1.27 (1.09 to 1.47); .002
Live birth	57/158 (36.1%)	76/160 (47.5%)	11.4 (0.6 to 22.2); .039	1.32 (1.01 to 1.72); .042	10.9 (−0.1 to 21.8); .053	1.30 (0.99 to 1.70); .059
Ongoing pregnancy	14/158 (8.9%)	18/160 (11.2%)	2.4 (−4.2 to 9.0); .480	1.27 (0.65 to 2.47); .482	1.6 (−5.1 to 8.4); .634	1.16 (0.56 to 2.41); .688
Complete to case sensitivity analyses						
Pregnancy	97/150 (64.7%)	126/156 (80.8%)	16.1 (6.2 to 26.0); .002	1.25 (1.08 to 1.44); .002	15.0 (5.0 to 24.9); .003	1.23 (1.07 to 1.42); .005
Live birth	57/150 (38.0%)	76/156 (48.7%)	10.7 (−0.4 to 21.8); .059	1.28 (0.99 to 1.66); .062	10.0 (−1.2 to 21.3); .081	1.26 (0.97 to 1.65); .088
Ongoing pregnancy	14/150 (9.3%)	18/156 (11.5%)	2.2 (−4.7 to 9.1); .529	1.24 (0.64 to 2.40); .531	1.4 (−5.6 to 8.4); .704	1.13 (0.55 to 2.32); .750
Outcomes among pregnant patients						
Live birth	57/97 (58.8%)	76/126 (60.3%)	1.6 (−11.5 to 14.6); .816	1.03 (0.82 to 1.28); .816	2.2 (−11.2 to 15.6); .753	1.04 (0.83 to 1.30); .748
Ongoing pregnancy	14/97 (14.4%)	18/126 (14.3%)	−0.1 (−9.5 to 9.2); .975	0.99 (0.52 to 1.89); .975	−0.8 (−10.7 to 9.1); .878	0.94 (0.46 to 1.90); .861
Pregnancy loss	26/97 (26.8%)	32/126 (25.4%)	−1.4 (−13.1 to 10.3); .814	0.95 (0.61 to 1.48); .813	−1.4 (−13.0 to 10.2); .815	0.95 (0.61 to 1.49); .838
Safety outcome						
Any adverse event	12/165 (7.3%)	12/165 (7.3%)	0.0 (−5.6 to 5.6); 1.000	1.00 (0.46 to 2.17); 1.000	/	/

*Data are presented as n/N (%) unless otherwise indicated. Effect estimates compare doxycycline plus MPA with doxycycline alone. Risk differences (RDs) and adjusted risk differences (aRDs) are expressed in percentage points. Adjusted estimates were obtained using the prespecified covariates described in the Methods. Treatment-by-baseline CE severity interaction analyses in the primary CE efficacy population did not provide evidence of effect modification on either the additive scale [difference in RDs, 4.6 percentage points (95% CI, −15.2 to 24.4); P for interaction = .650] or the multiplicative scale [ratio of RRs, 1.07 (95% CI, 0.82 to 1.40); P for interaction = .610]. No adjusted analysis was performed for adverse events. CE, chronic endometritis; CI, confidence interval; MPA, medroxyprogesterone acetate; RD, risk difference; RR, risk ratio; aRD, adjusted risk difference; aRR, adjusted risk ratio.

In the conservative full-cohort sensitivity analysis, in which patients who did not complete treatment or post-treatment pathological reassessment were classified as not cured, cure occurred in 130 of 165 patients (78.8%) receiving doxycycline plus MPA and 117 of 165 patients (70.9%) receiving doxycycline alone. The adjusted estimates were consistent with the primary analysis (aRD, 6.0 percentage points; 95% CI, −3.4–15.4; aRR, 1.08; 95% CI, 0.95–1.23) (Table 2).

In exploratory analyses stratified by baseline CE severity, cure occurred in 81 of 98 patients (82.7%) receiving doxycycline plus MPA and 75 of 96 patients (78.1%) receiving doxycycline alone among those with mild CE. Among those with severe CE, the corresponding cure rates were 49 of 62 (79.0%) and 42 of 62 (67.7%), respectively. The adjusted estimates and corresponding confidence intervals are presented in Table 2. Formal treatment-by-baseline CE severity interaction tests did not provide evidence of effect modification on either the additive scale (P for interaction = 0.650) or the multiplicative scale (P for interaction = 0.610) (Table 2).

### Safety outcomes

The safety analysis included all 330 patients who received treatment. Adverse events occurred in 12 of 165 patients (7.3%) in each group (RR, 1.00; 95% CI, 0.46–2.17) (Table 2). All adverse events were mild. The types and incidences are presented in Table 3.

**Table 3:** Adverse events according to treatment regimen.

Adverse event	Doxycycline (*N* = 165), n (%)	Doxycycline plus MPA (*N* = 165), n (%)
Any adverse event	12 (7.3)	12 (7.3)
Nausea or vomiting	12 (7.3)	9 (5.5)
Dizziness or headache	0	3 (1.8)

### Reproductive outcomes

The primary reproductive-outcome analysis included all 318 patients entering reproductive follow-up, comprising 158 patients receiving doxycycline and 160 receiving doxycycline plus MPA. Patients with incomplete follow-up were conservatively classified as not having experienced the corresponding outcome. A complete-case sensitivity analysis was conducted among the 306 patients with complete reproductive follow-up. Pregnancy occurred in 126 of 160 patients (78.8%) receiving doxycycline plus MPA and 97 of 158 patients (61.4%) receiving doxycycline alone. The adjusted estimates indicated a higher pregnancy rate in the doxycycline plus MPA group. (aRD, 16.5 percentage points; 95% CI, 6.5–26.5;   aRR, 1.27; 95% CI, 1.09–1.47; *P* = 0.002) (Table 2).

Live birth occurred in 76 of 160 patients (47.5%) receiving doxycycline plus MPA and 57 of 158 patients (36.1%) receiving doxycycline alone (aRD, 10.9 percentage points; 95% CI, −0.1–21.8; aRR, 1.30; 95% CI, 0.99–1.70). Ongoing pregnancy occurred in 18 of 160 patients (11.2%) and 14 of 158 patients (8.9%), respectively, with no clear evidence of a between-group difference (Table 2).

The complete-case sensitivity analysis of 306 patients with complete reproductive follow-up yielded results consistent with the primary analysis (adjusted RR for pregnancy, 1.23; 95% CI, 1.07–1.42). Among the 223 patients who achieved pregnancy and had an ascertainable pregnancy outcome, no clear between-group differences were observed in live birth, pregnancy loss, or ongoing pregnancy. The corresponding *P* values for the adjusted RRs were 0.748, 0.838, and 0.861, respectively (Table 2).

## Discussion

This prospective cohort study compared the efficacy, safety, and subsequent reproductive outcomes of doxycycline plus MPA versus doxycycline alone in women with RRF and CE. The addition of MPA did not demonstrate a clear improvement in histological CE cure after one treatment course but was associated with a higher subsequent pregnancy rate. No clear between-group differences were observed in live birth, miscarriage, or ongoing pregnancy, and both regimens were generally well tolerated.

CE is a disease characterized by disrupted homeostasis between the endometrial microbiota and the host immune system. Previous studies have shown that CE may adversely affect fertility and embryo implantation (5). The endometrial microbiota in CE has been reported to shift from Lactobacillus-dominant to non-Lactobacillus-dominant, accompanied by increased infiltration of immune cells within the endometrium, which contributes to the development of CE (32, 33). Therefore, restoration of the intrauterine environment forms the theoretical basis of CE treatment. Oral antibiotics are currently considered the most effective treatment in CE (25), and doxycycline monotherapy is the most widely used antibiotic regimen for CE (34). However, antibiotic eradication of potential pathogens alone may not be sufficient to fully reverse this dysregulated state. On the basis of anti-infective treatment, concomitant improvement of the endocrine and immune microenvironment related to endometrial receptivity may further improve reproductive outcomes. Limited evidence suggests that MPA may improve the local immune status of the endometrium (35), but its potential clinical benefit when combined with antibiotics for CE has not been established.

The improvement in CE cure was smaller than anticipated when the study was planned, with the adjusted effect being approximately half of the prespecified estimate. This difference may partly reflect the use of different progestogens, as the planning estimate was derived from a study of dydrogesterone, whereas the present study evaluated MPA (17–20). Differences in their pharmacologic profiles may lead to different endometrial responses. Nevertheless, the uncertainty surrounding the observed effect warrants cautious interpretation and further investigation.

The CE cure observed with doxycycline alone was broadly consistent with that reported in previous studies (17, 36). Although the cure rate was numerically higher with the addition of MPA, the present study did not provide clear evidence of an improvement in histological cure. MPA may influence the local immune microenvironment of the endometrium, and progesterone signaling contributes to endometrial secretory transformation and receptivity (19, 35). However, this potential mechanism was not directly evaluated in the present study and remains to be confirmed. Formal interaction tests did not provide evidence that baseline CE severity modified the association between treatment regimen and CE cure. Although cure appeared less frequent among patients with severe CE, the subgroup estimates were imprecise and should be interpreted cautiously. This finding may also partly explain the variability in antibiotic cure rates reported in previous studies, as diagnostic criteria for CE have differed across studies.

The addition of MPA was associated with a higher subsequent pregnancy rate despite no clear improvement in histological CE cure. This finding raises the possibility that histological CD138 negativity may not fully capture treatment-related changes in endometrial receptivity. Progesterone is involved in endometrial secretory transformation and local immune regulation and may therefore influence receptivity independently of histological cure (37). However, these mechanisms were not directly assessed, and residual confounding related to treatment selection and other reproductive factors cannot be excluded.

No clear between-group differences were observed in live birth, pregnancy loss, or ongoing pregnancy. These outcomes were assessed only among patients who achieved pregnancy and had an ascertainable pregnancy outcome, and the study was not specifically powered for these endpoints. The limited number of events reduced the precision of the estimates; therefore, type II error remains possible, and the absence of clear differences should not be interpreted as evidence of equivalence. Moreover, an association with pregnancy may not necessarily translate into a comparable improvement in live birth. Adverse-event incidence was similar between groups, and all reported events were mild (Table 3). These findings suggest that both short-course regimens were generally well tolerated in this study population.

This study has several limitations. First, treatment was selected during routine clinical care rather than randomly assigned; therefore, treatment-selection bias and residual confounding cannot be excluded, and the observed associations should not be interpreted as causal. Second, the sample size was calculated for histological CE cure rather than reproductive outcomes, and the limited number of reproductive events and incomplete follow-up reduced the precision of these estimates. Third, only one treatment course was evaluated at a single center, which may limit the generalizability of the findings. Larger multicenter studies and randomized controlled trials are required to confirm these findings.

## Conclusion

In women with RRF and CE, doxycycline plus MPA did not demonstrate a clear improvement in histological CE cure after a single treatment course compared with doxycycline alone but was associated with a higher subsequent pregnancy rate. No clear between-group differences were observed in live birth, pregnancy loss, ongoing pregnancy, or adverse event rates, and all reported adverse events were mild. Given the observational, single-center design, these findings should be confirmed in adequately powered multicenter randomized controlled trials.

## Data Availability

The de-identified data supporting the findings of this study are available from the corresponding author upon reasonable request, subject to institutional and ethical approval.
