# Supplementary material for: Clinical efficacy and pregnancy outcomes of doxycycline plus medroxyprogesterone acetate therapy for chronic endometritis in women with recurrent reproductive failure: a prospective cohort study
Source: Front Med (Lausanne). 2026 Sep 24;13:1913511. doi: 10.3389/fmed.2026.1913511 (PMC13645782; doi:10.3389/fmed.2026.1913511)
Supplement: Supplementary file 1 [file Table1.docx]

Supplementary Table S1 Analysis populations and missing outcome data by treatment group

| **Analysis population or data status** | **Doxycycline, n** | **Doxycycline + MPA, n** | **Total, n** |
| --- | --- | --- | --- |
| Enrolled population | 165 | 165 | 330 |
| Safety population | 165 | 165 | 330 |
| Not included in the primary CE efficacy analysis | 7 | 5 | 12 |
| Primary CE efficacy population | 158 | 160 | 318 |
| Complete reproductive follow-up | 150 | 156 | 306 |
| Incomplete reproductive follow-up | 8 | 4 | 12 |
| Primary reproductive-outcome analysis | 158 | 160 | 318 |
| Complete-case reproductive sensitivity analysis | 150 | 156 | 306 |
| Missing prespecified adjustment variables within the corresponding analysis populations | 0 | 0 | 0 |

^*^Of the 12 participants not included in the primary CE efficacy analysis, 7 did not undergo post-treatment hysteroscopy and endometrial biopsy, and 5 did not complete the prescribed treatment course. Participants with incomplete reproductive follow-up were conservatively classified as not having experienced the corresponding reproductive outcome in the primary analysis.
